# History of the Creation of Self-Gripping Mesh

**DOI:** 10.3389/jaws.2023.11330

**Published:** 2023-05-30

**Authors:** Maaike Vierstraete, Philippe Chastan, Alfredo Meneghin, Filip Muysoms

**Affiliations:** ^1^ Department of Surgery, Maria Middelares Hospital, Ghent, Belgium; ^2^ Retired, Lormont, France; ^3^ Retired, Trévoux, France

**Keywords:** inguinal hernia, hernia, mesh, ventral hernia, abdominal hernia

## Abstract

Self-gripping mesh (Progrip^TM^, Sofradim Production, Trévoux, France) was introduced in 2006 as a synthetic prosthetic material for reinforcement of the abdominal wall in open inguinal hernia repair. As of September 2022, the self-gripping mesh has been implanted 4 million times. In June 2014 at the annual Mesh congress in Paris during an informal conversation with Dr. Chastan, Dr. Muysoms became intrigued by the history of the invention and creation of this self-gripping mesh. His fascination on this topic, was the initial bead implanted for this project to write down the history of the creation of self-gripping mesh.

## Introduction

Self-gripping mesh (Progrip^TM^, Sofradim Production, Trévoux, France) was introduced in 2006 as a synthetic prosthetic material for reinforcement of the abdominal wall in open inguinal hernia repair. It arose as an alternative for an open Lichtenstein mesh repair, to avoid the use of a non-adhesive mesh that had to be reapproximated with sutures after encircling the cord structures. Since its introduction, the self-gripping mesh has also been introduced for the repair of groin hernias via a laparoscopic approach as well as the repair of ventral hernias or suture line reinforcement. As of September 2022, self-gripping mesh has been implanted 4 million times. In June 2014 at the annual Mesh congress in Paris during an informal conversation with Dr. Chastan, Dr. Muysoms became intrigued by the history of the invention and creation of a self-gripping mesh. His fascination on this topic, was the initial bead implanted for this project to write down the history of the creation of self-gripping mesh.

## Methods

On 26th October 2022, a one-day meeting was organised in Trévoux, France at the Sofradim Production Research and Development centre to interview Philippe Chastan, the surgeon, and Alfredo Meneghin, the textile engineer, who were both involved in the creation of the self-gripping mesh from the beginning until it was introduced for clinical use. Both are currently retired but very enthusiastic to take part in this project. The day also included an on-site visit to the manufacturing centre to learn and see how the self-gripping mesh is produced. Written informed consent was obtained for identifiable images included in this article.

### The Prehistory

**FIGURE 1:**
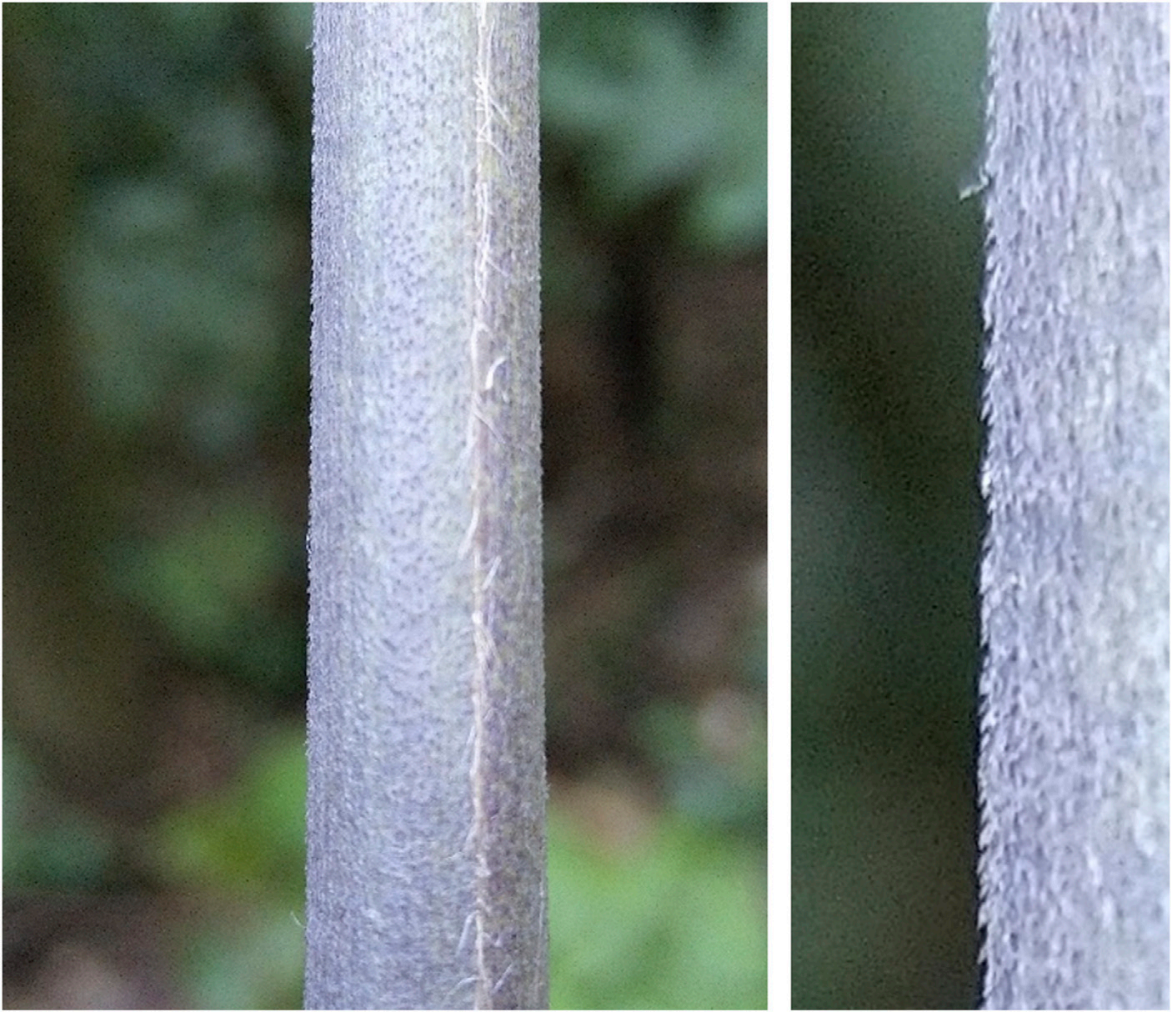
An example of grips produced by plants in the Brazilian tropical woods to achieve mechanical adhesion to other surfaces.

In 1955 George de Mestral, a Swiss engineer and inventor, patented the invention of Velcro®, a way to create adhesion between fabrics [1]. The idea came during a hike when his dog was covered with burr seeds of the big burdock plant (Arctium lappa) that had attached firmly to the dog’s fur. Closer examination learned that minuscule hooks were responsible for the attachment of the burr to various structures. By recreating fabrics with a similar hook-to-hook or hook-to-loop configuration, the fabrics were able to stick together in the same way the burr seeds had attached to the dog’s fur. In nature, there are several well-known examples of mechanical adhesion by minuscule hooks, for example “Le chardon naturel” (the thistle) being a well-known example and a tropical Brazilian plant where the stem is covered with tiny grips making it sticky to the surfaces of other structures (Figure 1).

**FIGURE 2:**
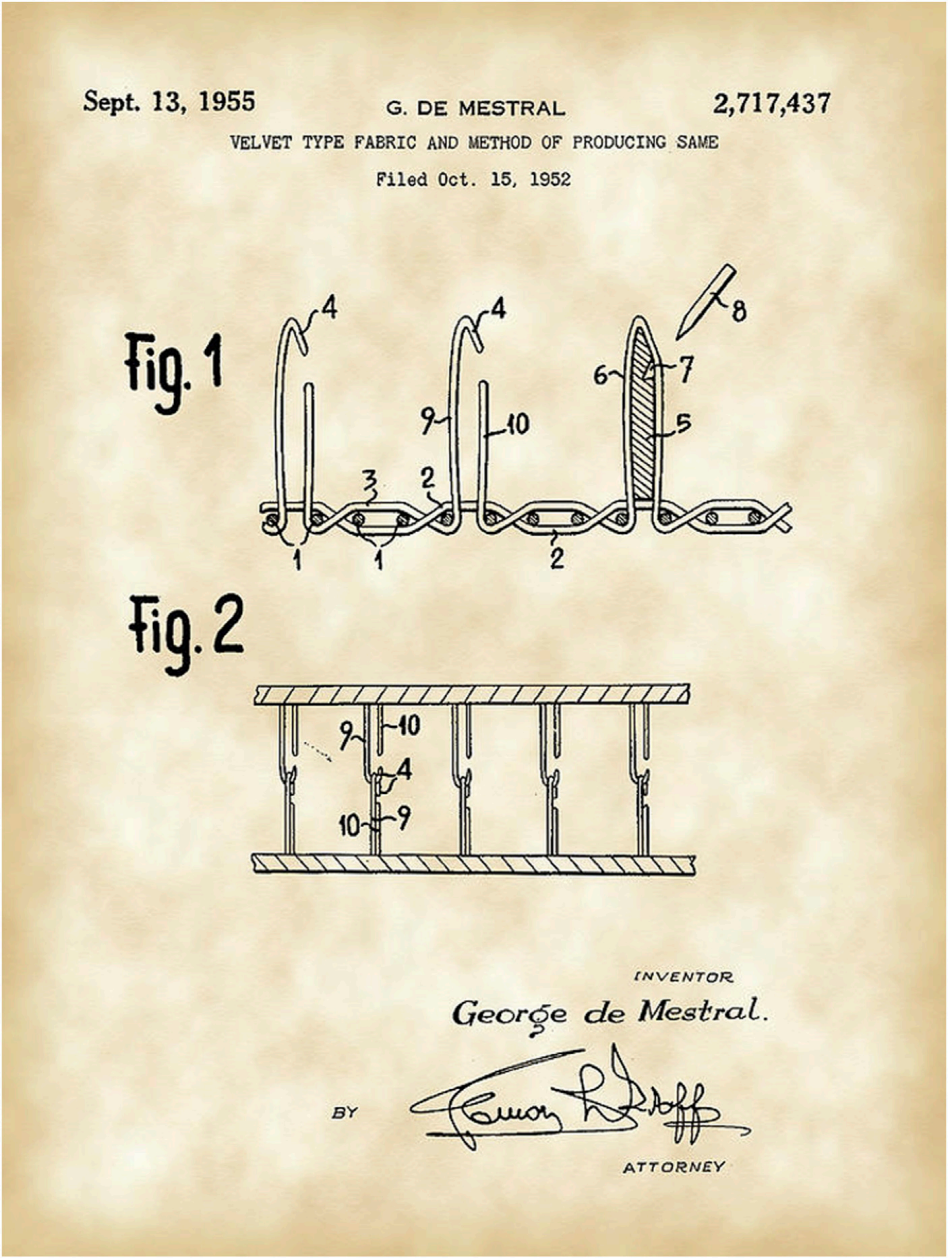
Patent for the concept of creating Velcro® by George de Mistral a Swiss engineer and inventor [1].

George de Mistral created the small hooks by cutting small woven loops of yarns on each side of the fabrics so the hooks could grip into each other resulting in adhesion between two fabrics. Initially it was not easy to find the ideal material with enough memory and stability for a durable fixation. After testing several materials in Lyon, France, he discovered nylon, a polyamide synthetic polymer, to have the right characteristics needed to create the hook-to-hook configuration. This technique was patented in 1955 in the United States. Figure 2 shows a picture of the original patent. The name Velcro® is derived from the French words “Velours” (velvet) and “Crochet” (hook) and it became popularised after NASA used the technology for the space suits of their astronauts.

To be adhesive, one side should have small hooks or grips and the other side needs to have either small loops (hook-to-loop configuration) or also small hooks (hook-to-hook configuration) for the grips to get attached. Alfredo Meneghin refers to these as the “male side” (hooks) and the “female side” (loops). He worked previously in the car industry to create strong and reliable adhesions between several fabrics of car seats. He also worked in the production of terry cloth where loops were made in the textile to give the cloths a softer surface and feeling. In 1997 he started working for Sofradim as responsible lead for research and experimentation in producing textiles that could be used for repair of abdominal wall hernias. The centre is located in Trévoux, France. This town, on the borders of the river Saône, is known since the thirteenth century for its skills in dies and extrusion, a process where materials are pulled into small diameter yarns thus creating the appropriate material for weaving and knitting. As they were also highly skilled in making gold yarns the town came to a high level of prosperity which created a very wealthy city that was at one point an independent state within France. As a result, the region of Trévoux and Lyon has had a tradition in textile engineering and production of both raw materials (polyester, polypropylene), yarn production and the medical industry making sutures or meshes.

### Discovery of Grips

**FIGURE 3:**
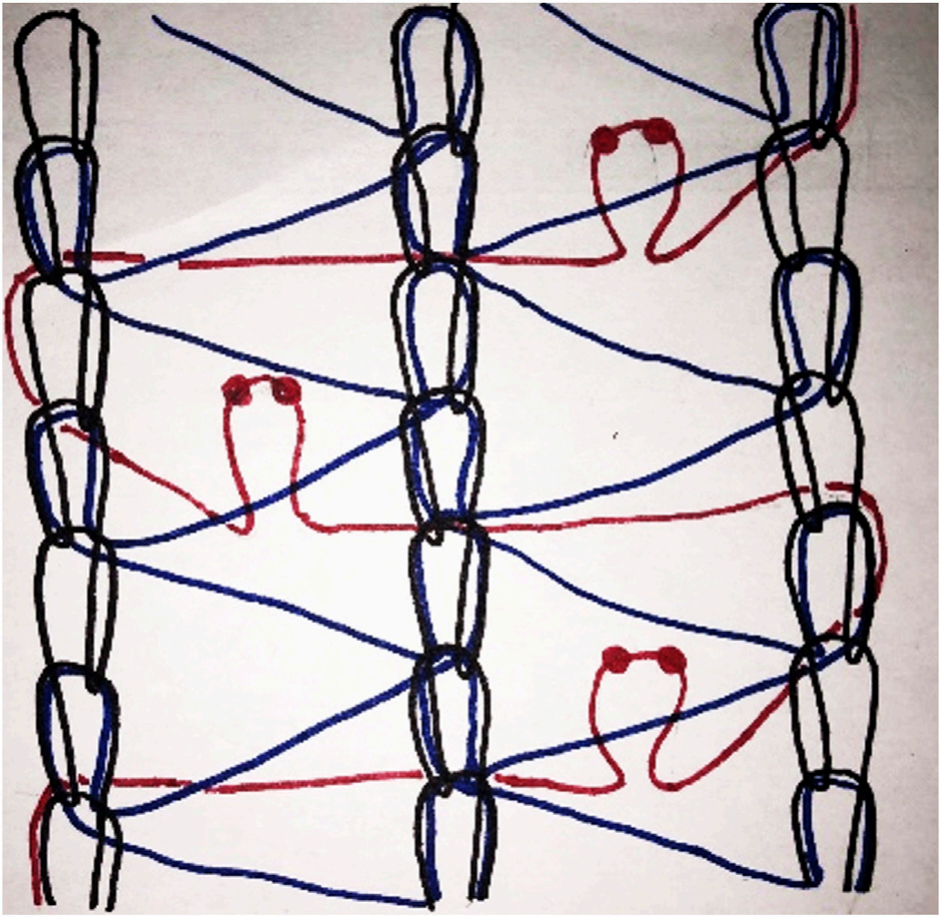
A diagram of a knitted construction of Progrip prototype; polyethylene terephthalate (PET, black and blue yarn), polypropylene (PP, red yarn).

**FIGURE 4:**
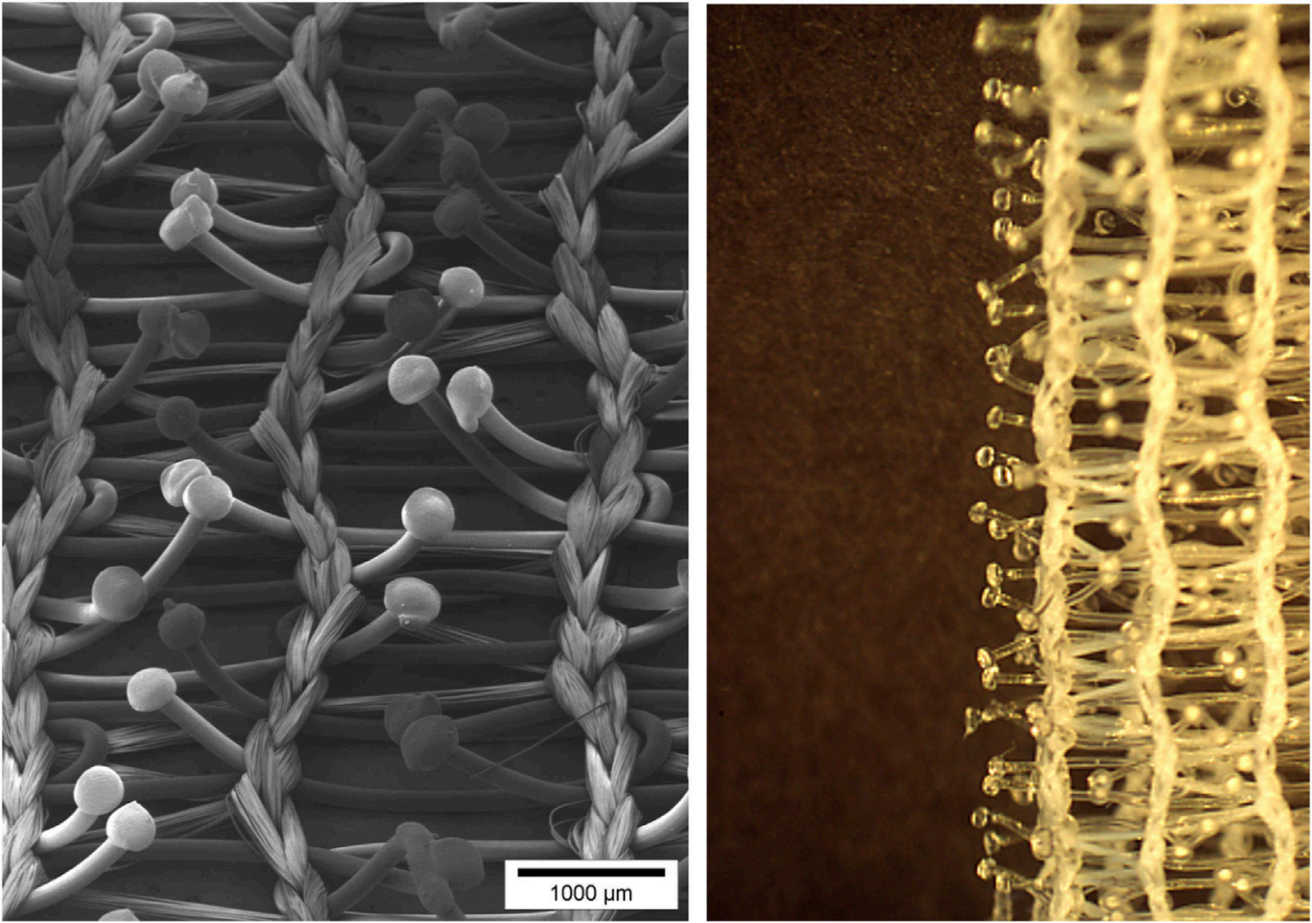
Mushroom-like grips are produced by heating yarns of the loops of the knitted fabric.

With all the previous experience, a warp-knitted mesh of multifilament polyethylene terephthalate (PET—usually referred to as polyester) mesh was created with a superimposed monofilament polypropylene (PP) yarn creating a loop configuration on the surface of the mesh. A diagram of how such a knitted construction looks like is given in Figure 3. The challenge is to create a stable construction as the loop-yarn should not come loose from the bottom mesh once the yarns are cut. Also, the technology should be able to deliver a standardized and homogenous final mesh product with no or minimal variability between the end products delivered to the surgeon for implantation. While experimenting with the mesh, Alfredo Menighen wanted to test if the loops of PP would turn black when exposed to heat with a soldering iron. While rubbing the soldering iron on the mesh surface, the polypropylene did not change colour, but the loops got interrupted creating for each loop two separate grips that looked like tiny mushrooms (Figure 4). The grips were created and formed the “male” side that could attach to the “female” side of mesh surface that was not exposed to heat of the rubbing soldering iron. Thus, a mesh was created that could be fixed to another mesh.

### Surgeons Input

**FIGURE 5:**
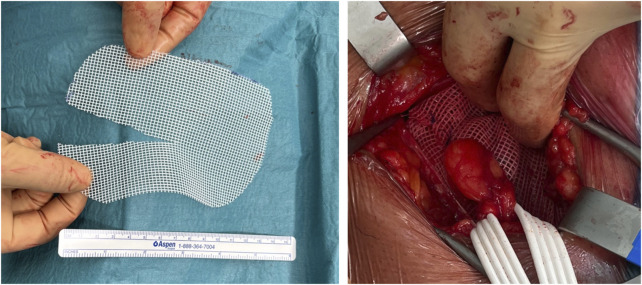
Intraoperative picture of a traditional Lichtenstein mesh (Parietex™ Hydrophilic 2-Dimensional Mesh) with a lateral slit for the cord and sutures placed lateral of the cord to join the flaps.

With the discovery and the concept of grips allowing to fix mesh on mesh, Alfredo Menighen went to the surgeon Dr. Chastan to discuss if he could see any use for this concept in surgical practice. At that time Dr. Chastan was using the Lichtenstein technique for open mesh repair in most patients with groin hernias. This technique involves the reinforcement of the posterior wall of the inguinal canal with a mesh on top of the fascia transversalis. The mesh is traditionally fixed with a running suture to the inguinal ligament at its lower border besides some interrupted sutures to the falx inguinalis at its upper border. Laterally, a slit is made in the mesh to allow the inguinal cord to pass through the mesh at the medial end of the slit. The two flaps are then sutured to each other to close the slit creating a hole in the mesh for the cord (Figure 5).

**FIGURE 6:**
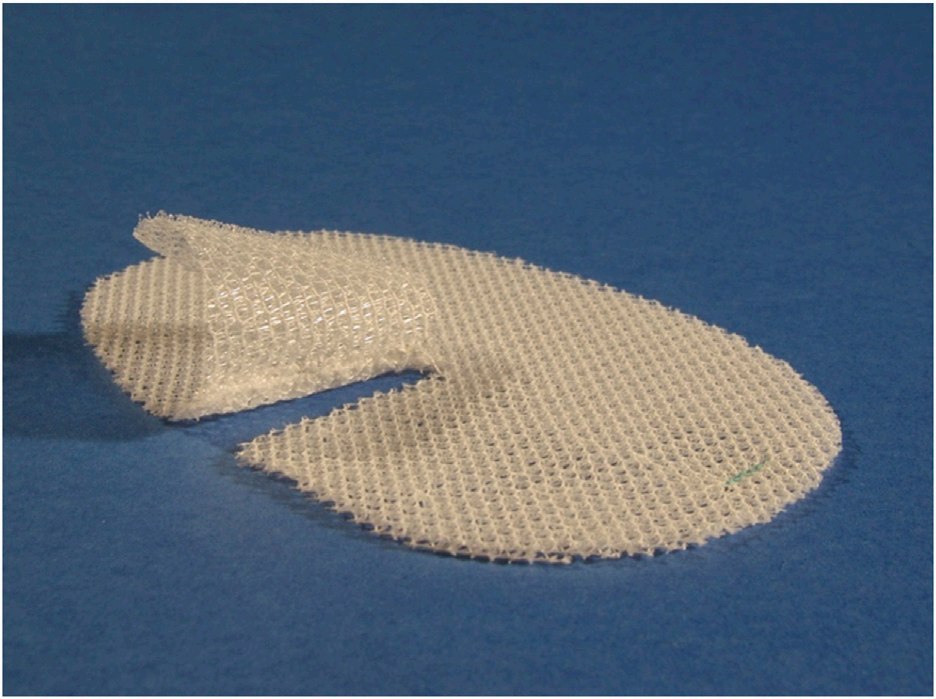
Parietex® Easegrip® mesh for groin hernia repair in the “Lichtenstein” position, left sided version. A small marker indicates the medial side of the mesh, and the flap is cranial.

**FIGURE 7:**
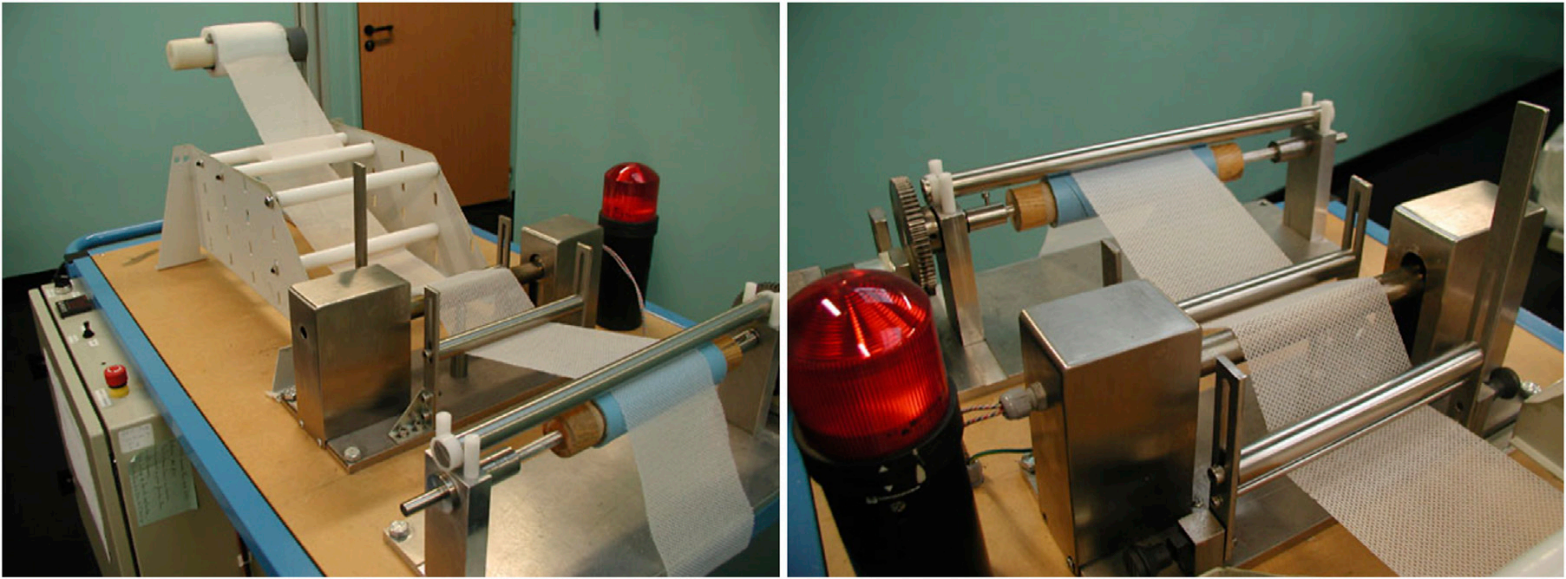
The prototype machine that Alfredo Meneghin built in 1998 to produce a self-gripping mesh.

Dr. Chastan came up with the idea to create a flap on top of a “Lichtenstein” mesh to close the slit, thus omitting the need for additional sutures to close the slit. This idea led to the development of Parietex® Easegrip®. The flap is covered with grips (male side) that can be attached to the underlying mesh (female side) (Figure 6). The prototype machine that Alfredo Meneghin built in 1998 to procedure the flap part with grips is shown in Figure 7.

Dr. Chastan performed a study in 30 patients intraoperatively to measure how big a mesh should be to adequately cover the inguinal region for repair. The measurements were remarkable homogeneous amongst patients of different gender, different body mass index and other variables. A mesh of 10 cm in width and 6 cm in height would cover the inguinal area sufficiently. It was decided to make the mesh 12 cm × 8 cm, in a left and right-sided anatomical version. This additional 2 cm allowed to overlap the inguinal ligament by 2 cm. Ultimately also a larger mesh of 14 cm × 9 cm was provided and launched because for many surgeons these are the dimensions, they preferably use for their Lichtenstein repairs.

### Production of Self-Gripping Mesh

**FIGURE 8:**
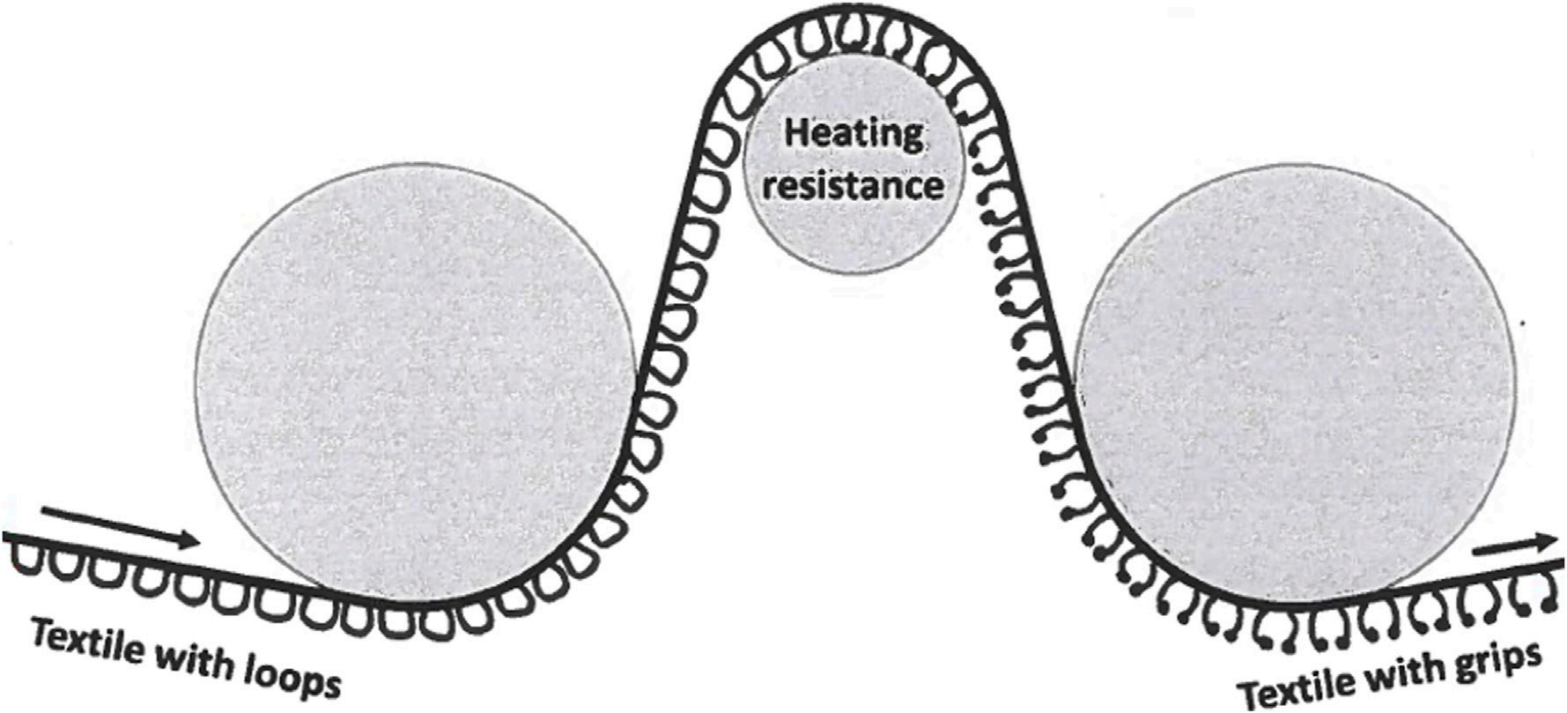
The loops of the mesh will melt partially and create the mushroom-like grips when the mesh is run over the heated bar.

The textile with loops is brought in contact with a bar of stainless steel inside of which there is an electronic resistance that can produce intense heat of a temperature up to 200°C. The loops of the mesh will melt partially and create the mushroom-like grips when the mesh is run over the heated bar (Figure 8). There are a lot of variables that will influence the extent of “melting” of the loops and the final configuration of the grips in length and volume of the “mushroom”. First, the speed with which the mesh is running over the heated bar will determine the time of exposure of the loops to the heat. Also, the temperature produced by the resistance can be variable and will determine the outcome of the grips. Finally, the distance of the mesh to the heated bar will play its role.

## An Unexpected Finding and its Consequences

While Dr. Chastan was testing and using Parietex® Easegrip®, the grips of the flap got attached to the cremasteric muscle of the cord. Apart from this being a bit annoying, it also made Dr. Chastan realize the potential for the grips to attach to natural tissues as muscle or fascia. He contacted Alfredo Meneghin asking him if he would be able to produce a mesh completely covered with grips over the whole surface of the mesh. This might obliviate the need for all suture fixation to the inguinal ligament and to the falx inguinalis in a traditional Lichtenstein repair.

Thus, Alfredo Meneghin started to produce small patches of mesh of 6 cm × 6 cm made of PP mesh with grips in PP which he provided to Dr. Chastan for evaluation on their capacity to stick to muscular tissue. Overall, about 50 tests were done to find the most appropriate setting of the production variables with the best gripping strength. Another variable that will influence the gripping capacity is the density of the loops (number of loops per square cm).

### Concerns About Non-Absorbable Grips

**FIGURE 9:**
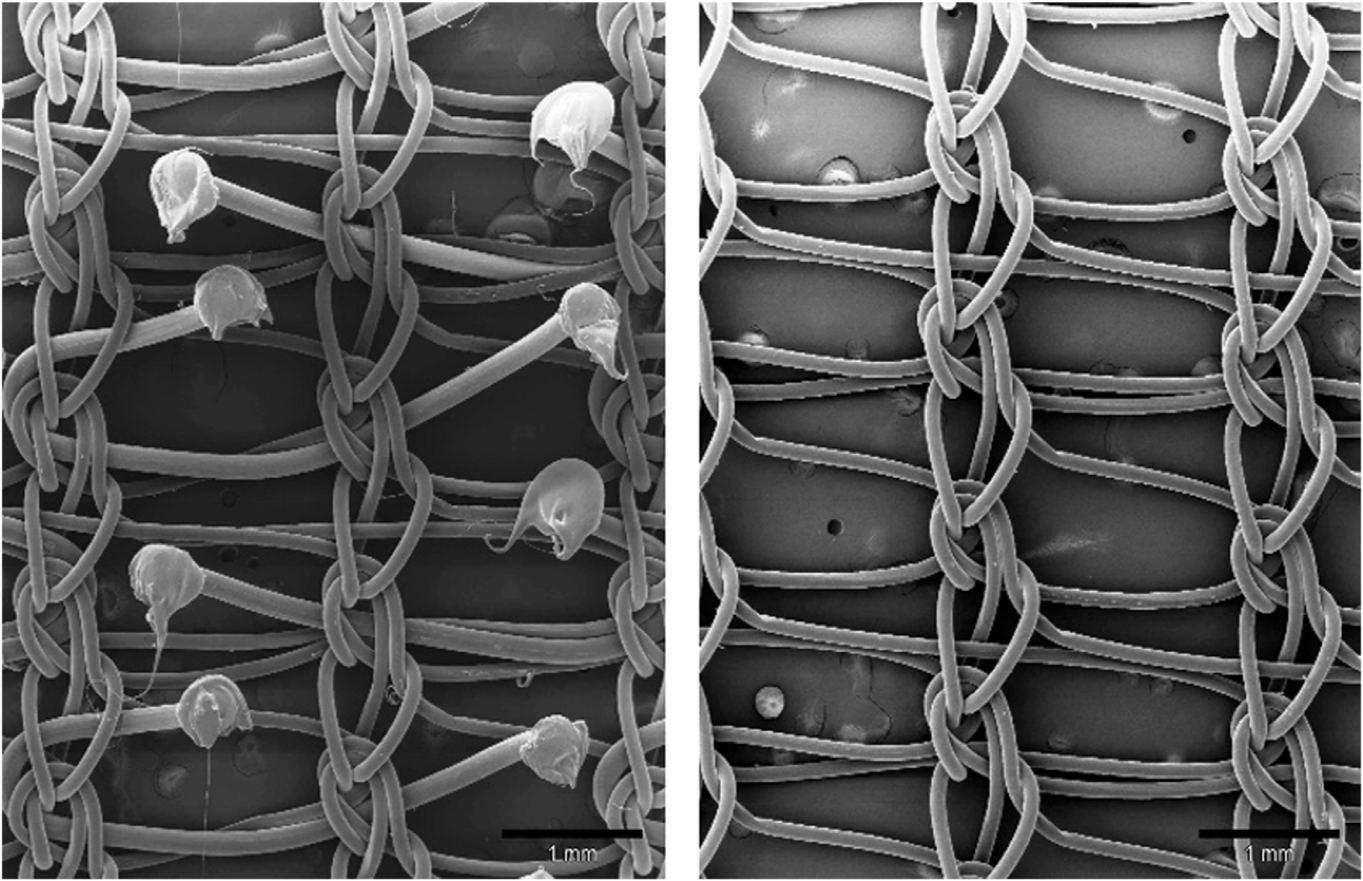
Picture of Progrip™ Self-Gripping Polypropylene (Pg-PP) mesh before and after resorption of the PLA grips.

For these first trials, the grips were made of non-absorbable PP which led to major concerns about possible adverse effects of the non-absorbable mesh contact with the inguinal nerves. For this reason, the project was re-scoped. After some further experimentation Alfredo Meneghin found a new monofilament yarn made of absorbable material that could be used and had the right properties to provide grips in a standardized manner. The yarn was produced from polylactic acid (PLA) which is essentially degraded in 3–4.5 years after implantation (Figure 9). The weight of the Progrip™ Self-Gripping Polypropylene (Pg-PP) mesh before PLA resorption is 77 g/m^2^ and after resorption 43 g/m^2^.

### From Polypropylene to PET

The mesh Pg-PP was launched in early 2006 with a limited release. In a prospective study with 2 years of follow up, Dr. Chastan performed 50 mesh implants in the Lichtenstein position. One absorbable suture was placed on the pubic bone to avoid the mesh to shift laterally during the placement of the mesh. The latter to avoid exposing the medial area of the groin uncovered, being a possible site of recurrence. The fascia transversalis was not sutured to adhere to the principles of a tension-free groin hernia repair. With these positive initial clinical results, the Pg-PP mesh was commercialized. The results of the study were later published in the Hernia journal in 2009 as a prospective series of 52 patients with 70 implants and 12 months follow up [2]. Later, Sofradim Production had built solid evidence on PET safety, efficiency, and biocompatibility, both in bench tests and preclinical models. They introduced Pg-PET mesh, where the polypropylene monofilament yarns were replaced by PET monofilament yarns, still combined with the PLA absorbable grips. Simultaneously, an industrial machine enabling to perform the gripping process was developed.

### Expanding Mesh Indication to Ventral Hernia Repair and Hernia Prevention

Pg-PP mesh was fully released in 2007, providing preformed meshes and 15 cm × 9 cm flatsheet meshes for inguinal hernia repair via the anterior tension-free approach. Pg-PET mesh was initially released in 2008, providing preformed meshes and flatsheet (15 cm × 9 cm and 15 cm × 15 cm) meshes indicated for inguinal and incisional hernias repair. Later, in 2011, larger meshes up to 30 cm × 15 cm were created and commercialized. Hopson et al. [3] and Kroese et al. [4] reported promising results of the retromuscular placement of the Pg-PET mesh in ventral hernia repair. Pg-PP mesh indication was further expanded in 2018 for abdominal suture line reinforcement in the prevention of incisional hernias.

### Laparoscopic Progrip

Although not intended to be used for laparoscopic surgery some surgeons started using the flat Pg-PET mesh during laparoscopic groin hernia repairs. Probably, Dr. Dieter Birk was one of the first surgeons starting to use Pg-PET mesh in laparoscopy in 2008. Favourable results during a follow-up period of 23 months were published in 2013 [5]. Muysoms et al. also started using Pg-PET mesh in laparoscopic groin hernia repairs in November 2009 [6]. Sofradim Production later produced a mesh dedicated for laparoscopic groin hernia repair, Progrip™ Laparoscopic Self-Fixating Mesh. This mesh has a fast-resorbing collagen film to allow easier unfolding of the mesh without too much mesh-to-mesh adhesion during implantation. There is also an anatomical version manufactured to accommodate for the specific anatomy of the groin. After the production of the Pg-PET mesh of 30 cm × 15 cm, Muysoms et al. started in 2010 using a one single mesh of 28 cm × 13 cm to cover both myopectineal orifices in laparoscopic bilateral preperitoneal hernia repair [7].

## Future Needs

Surgeons have been asking for several years for wider self-gripping meshes with dimensions of 30 cm × 30 cm or even bigger for large ventral hernia repairs. The issue is that it is not easy to produce meshes wider than 15 cm with enough homogeneity over the whole width of the mesh. And of course, this is a requisite for a medical device to be reproducible and of reliable homogeneity. Therefore, until today all self-gripping meshes are limited to a width of 15 cm maximally. However, Sofradim Production is currently improving their manufacturing capabilities to allow the production of wider meshes.

## Data Availability

The original contributions presented in the study are included in the article/supplementary material, further inquiries can be directed to the corresponding author.
